# Combined assessment of ERO1A expression and CD163^+^ tumor-associated macrophage infiltration is superior to traditional assessment methods in predicting clear cell renal cell carcinoma prognosis

**DOI:** 10.3389/fonc.2026.1732415

**Published:** 2026-02-23

**Authors:** Tianyu Hong, Changjie Ren, Hao Xiang, Huaye Wu, Yongwei Yu, Mierxiati Abudurexiti, Zhong Wang, Chao Wang

**Affiliations:** 1 School of Gongli Hospital Medical Technology, University of Shanghai for Science and Technology, Shanghai, China; 2 Department of Urinary Surgery, Pudong Gongli Hospital, Shanghai University of Medicine and Health Sciences, Shanghai, China; 3 The Third Affiliated Hospital of Zunyi Medical University, The First People’s Hospital of Zunyi, Zunyi, Guizhou, China; 4 Department of Obstetrics and Gynecology, Chengdu Women’s and Children’s Central Hospital, School of Medicine, University of Electronic Science and Technology of China, Chengdu, Sichuan, China; 5 Department of Pathology, Changhai Hospital, Naval Medical University, Shanghai, China; 6 Shanghai Health Commission Key Lab of Artificial Intelligence-Based Management of Inflammation and Chronic Diseases, Gongli Hospital of Shanghai Pudong New Area, Shanghai, China

**Keywords:** CD163, clear cell renal cell carcinoma, ERO1A, prognosis, tumor-associated macrophages

## Abstract

**Purpose:**

The interplay between cancer cells and tumor-infiltrating immune cells (TIICs) facilitates the development of clear cell renal cell carcinoma (ccRCC). However, it remains unclear whether the combined assessment of tumor biomarkers and TIIC density increases the accuracy of ccRCC prognosis prediction.

**Methods:**

ERO1A expression was detected in ccRCC tissue samples and cell lines. ccRCC patients from two hospitals were recruited to investigate the value of ERO1A and other TIIC biomarkers in determining ccRCC prognosis via immunohistochemistry (IHC) and statistical analyses.

**Results:**

Compared with that in the corresponding controls, ERO1A expression in ccRCC cell lines and tissues was increased and closely correlated with ccRCC progression. ERO1A expression was positively correlated with the density of infiltrating CD163^+^ tumor-associated macrophages (TAMs) in ccRCC. Among all groups of patients, ccRCC patients with high expression of both ERO1A and CD163 presented the highest TNM stage or Mayo Clinic stage, size, grade, and necrosis (SSIGN) score, as well as the shortest survival period. Moreover, the independent risk factors for the survival of ccRCC patients included ERO1A expression, CD163^+^ TAM density, TNM stage, and the SSIGN score. Time-dependent C-index analysis revealed that the combination of ERO1A expression and CD163^+^ TAM density was superior to either factor alone in predicting ccRCC patient survival. Furthermore, the combination of ERO1A expression and CD163^+^ TAM density with TNM stage or the SSIGN score had the greatest ability to predict the survival of ccRCC patients.

**Conclusions:**

Combining ERO1A expression, CD163^+^ TAM density, and TNM stage or SSIGN score increases the accuracy of postoperative survival prediction for ccRCC patients.

**Précis:**

Compared with currently available biomarkers, the combination of ERO1A expression and the density of infiltrating CD163^+^ TAMs with TNM stage or the SSIGN score exhibits greater accuracy in evaluating ccRCC patient survival.

## Introduction

Clear cell renal cell carcinoma (ccRCC) is among the most common malignant tumors worldwide and accounts for many new cancer cases and related deaths (1). Although patients with localized ccRCC typically undergo surgical treatment, the 5-year overall survival rate remains low (2). Therefore, it is necessary to identify reliable indicators for postoperative monitoring of ccRCC patients to help in clinical decision making and improve the survival rate of ccRCC patients (3). In general, the tumor–node–metastasis (TNM) staging system is currently widely used treatment decision making for ccRCC patients (4). However, TNM staging has limitations in accurately assessing ccRCC prognosis.

Many studies have recently revealed that tumor biomarkers, such as AURKB and ANGPTL4, can serve as prognostic factors for ccRCC (5, 6). Our previous studies have demonstrated that ccRCC patients with high gankyrin expression and low SOX17 expression have short survival periods (7, 8). Additionally, the oncogenic roles of long noncoding RNAs (lncRNAs), including LINC02609 and LINC00926, have recently been recognized as useful indicators of relapse and progression risk in ccRCC patients (9, 10). However, many research centers use a single biomarker to evaluate the survival and disease progression of ccRCC patients, which is largely a one-sided approach. Therefore, integrated prognostic indices, such as stage according to the University of California–Los Angeles Integrated Staging System and the Mayo Clinic stage, size, grade, and necrosis (SSIGN) score, have been developed (11). Although these studies show promise, additional validation is needed in basic science and clinical studies because these prognostic indices reflect only the features of ccRCC tissues and not those of the tumor microenvironment.

Tumor recurrence and metastasis are related to the internal factors of the tumor and the tumor microenvironment (12). Both are equally important and cannot be ignored. Numerous studies, including our previous study (13, 14), have shown that tumor-associated macrophages (TAMs), a key type of tumor-infiltrating immune cell (TIIC) in the cancer microenvironment, are regulated by cancer cells and facilitate cancer development. Furthermore, CD163 has been recognized as a TAM marker, and CD163^+^ TAMs can be utilized as a prognostic marker for ccRCC; these cells may also be therapeutic targets for cancer immunotherapy (15). However, studies on whether combining cancer biomarkers, TAM infiltration, and clinical indicators increases the accuracy of assessing ccRCC patient survival are lacking. Thus, the present study aimed to assess the value of integrating endoplasmic reticulum oxidoreductase 1 alpha (ERO1A) expression and CD163^+^ TAMs with TNM stage or the SSIGN score in predicting ccRCC patient survival.

ERO1A (also named ERO1α) is an endoplasmic reticulum (ER)-resident, thiol oxidoreductase-containing flavin adenosine dinucleotide (FAD) that is responsible for catalyzing the formation of disulfide bonds in newly synthesized peptides, and it functions synergistically with protein disulfide isomerase (PDI) (16). The dysregulation of oxidative protein folding mediated by ERO1A has been mechanistically linked to multiple oncogenic pathways. Its overexpression not only sustains tumor cell proliferation under hypoxic conditions but also promotes epithelial-mesenchymal transition (EMT), angiogenesis, and evasion of immune surveillance through redox-dependent mechanisms (17, 18). Furthermore, ERO1A-mediated oxidative stress has been shown to activate HIF-1α and NF-κB signaling pathways, thereby fostering tumor adaptation to metabolic stress and inflammatory responses (19). These molecular insights substantiate ERO1A’s emerging role as a critical node connecting proteostasis, tumor metabolism, and immune evasion in cancer progression. ERO1A has recently been reported to be upregulated in tumors and to be involved in tumor growth, metastasis and chemoresistance (16). Furthermore, ERO1A has been reported to modulate the immunosuppressive tumor immune microenvironment (20). However, it remains unclear whether ERO1A expression can be used to assess ccRCC patient disease progression and prognosis.

In the present study, we assessed the value of combining ERO1A expression and the CD163^+^ TAM density in assessing ccRCC patient survival. In particular, we assessed whether the integrated assessment of ERO1A expression and the CD163^+^ TAM density with TNM stage or SSIGN score is superior to the assessment of TNM stage or the SSIGN score alone for predicting disease progression and survival in ccRCC patients.

## Materials and methods

### Analysis of ccRCC samples from the cancer genome atlas

RNA-seq information for kidney renal clear cell carcinoma (KIRC) was obtained from TCGA. The gene expression information was obtained in TPM format; data from 613 samples (479 unpaired tumor samples, 72 unpaired normal samples, and 72 paired tumor and adjacent normal samples) were included. The expression levels were log2-transformed [log2(TPM + 1)]. The Wilcoxon rank-sum test and paired t test were individually applied to compare the expression of ERO1A between unpaired ccRCC samples and corresponding normal samples or between paired ccRCC tissues and normal samples.

### Clinical data of ccRCC patients

A total of 406 patients who were pathologically diagnosed with ccRCC from two clinical units (cohort 1, n=82; Shanghai Pudong New Area Gongli Hospital; cohort 2, n=324; Shanghai Changhai Hospital) were included in this study. Two pathologists conducted double-blinded clinicopathological data analysis on all ccRCC patients and independently scored all ccRCC samples. In addition, 68 pairs of ccRCC tissue samples were subjected to real-time PCR experiments. The present study strictly followed the Reporting Recommendations for Tumor Marker Prognostic Studies (REMARK) (21). A summary of the clinicopathological features of the enrolled ccRCC patients is presented in **Supplementary Table S1**. The ccRCC patients (n=406) from the two clinical centers were randomly divided into training (n=203) and validation (n=203) cohorts. The primary outcomes of ccRCC patients were overall survival (OS) and progression-free survival (PFS). All the assays were authorized by the Institutional Ethical Review Board of Shanghai Gongli Hospital and Changhai Hospital, and written informed consent was obtained from the ccRCC patients.

### Real-time PCR assays

Real-time PCR experiments were conducted as previously described (8). Total RNA from the cell lines was extracted with TRIzol (Gibco, 15596018). cDNA was synthesized using the PrimeScript One Step RT Reagent Kit (Takara, RR064A). Quantitative RT–PCR was conducted with SYBR Green Real-Time PCR Master Mix (Toyobo, QPK201) and a StepOnePlus Real-Time PCR System (Applied Biosystems). The experimental results were normalized to the expression of glyceraldehyde 3-phosphate dehydrogenase (GAPDH). The 2^-△△Ct^ method was applied to calculate the expression level of each target gene relative to that of the control.

The following primer sequences were used: *ERO1A* (forward primer, 5’-TTGGATCTGCTGGTGGTCAT-3’; and reverse primer, 5’-CCAGTGTAGCGCTCAGGATT-3’) and GAPDH (forward primer, 5’-TGGCACCGTCAAGGCTGAGAA-3’; and reverse primer, 5’-TGGTGAAGACGCCAGTGGACTC-3’).

### Immunohistochemistry experiments

IHC experiments were performed as previously reported (8). In brief, the samples were fixed in 4% methanol, embedded in paraffin, and subsequently cut into 5-µm-thick sections. Deparaffinization and rehydration were performed according to routine methods, and antigen recovery was performed in heated citrate buffer (pH 6.0) or EDTA buffer (pH 8.0) for 30 minutes. The tissue microarray slides were processed according to the UltraSensitive SP (Mouse/Rabbit) IHC Kit (KIT-9710; Maixin Biotechnologies). Specifically, each slide was incubated with endogenous peroxidase blocking solution for 30 minutes, and nonspecific binding sites were blocked with normal animal nonimmune serum for 20 minutes.

The slides were incubated with the following primary antibodies at 4 °C overnight: rabbit anti-ERO1A antibody (1:100; ab177156; Abcam), rabbit IgG antibody (1:100; Cell Signaling Technology), rabbit anti-CD163 antibody (1:500; GB115709; Servicebio), mouse anti-FOXP3 (1:200; ab2004; Abcam), and rabbit anti-CD8 (1:200; ab4055; Abcam). The slides were subsequently incubated with a biotin-labeled secondary antibody and Streptomyces antibiotic protein-peroxidase for 30 minutes. Afterward, diaminobenzidine (DAB) (DAB-2031; Maixin Biotechnologies) was used for staining.

The sections were subsequently incubated with hematoxylin and assessed by two pathologists in a double-blinded manner. ERO1A staining was scored according to the H-score approach; the H-score was calculated by multiplying the fraction of each component observed in the sample sections (as calculated by the intensity score [range 0–3] by the percentage of positive cells [range 0–100], with a total score range of 0–300) (8). The whole slide was first observed under a low-power microscope (40× or 100× magnification). Three representative fields of view were randomly selected under a high-power microscope (200× magnification) and scored according to the previous grading method. The mean value was subsequently calculated. For TIICs, the whole slide was first observed at ×40 or ×100 magnification. Three randomly selected areas of ccRCC were subsequently evaluated at ×200 magnification to score the density of stained TIICs. Finally, the mean value was calculated. The total cell count was calculated as the number of nucleated dyed cells per field and is presented as the density (cells/mm^2^) (8).

### Cell culture and RNA interference

The cell lines were purchased from the Cell Bank of the Type Culture Collection of the Chinese Academy of Sciences in 2023. A498 and ACHN cells were cultured in minimum essential medium (Gibco, 11095-080), and 786-O cells were cultured in RPMI-1640 medium (Gibco, 22400-089). HK-2 cells were cultured in high-glucose Dulbecco’s modified Eagle’s medium (DMEM) (Gibco, 11995-065). The culture media of the cell lines were supplemented with fetal bovine serum (FBS, 10%, Gibco) and 1% penicillin/streptomycin (Gibco). The cell lines were incubated at 37 °C in 5% CO_2_. Sunitinib- and pazopanib-resistant 786-O cells were cultured in RPMI-1640 medium supplemented with 10% (v/v) FBS and 10 µM sunitinib or 8 µM pazopanib. All cell lines were authenticated by short tandem repeat (STR) profiling and examined for mycoplasma contamination via a Mycoplasma detection kit (Selleck Chemicals), and the most recent tests were completed in April 2024. The cell lines were used within 40 passages.

RNA interference was carried out following the manufacturer’s instructions using Lipofectamine™ RNAiMAX Transfection Reagent (Invitrogen). In brief, siRNA directed against ERO1A and a non-targeting control siRNA (obtained from Sangon Biotech (Shanghai, China) with sequences: si-ERO1A−1: 5’- GAAGGCTGTTCTTCAGTGGACC-3’; si-ERO1A −2: 5’- CCCTTGTAACCAGTGTAGCGCT-3’.) were prepared in RPMI 1640 Reduced Serum Medium. The diluted siRNA was combined with Lipofectamine™ RNAiMAX reagent and allowed to incubate at room temperature for 20 minutes to allow complex formation. This mixture was subsequently added to the cell cultures in a dropwise manner. Following a 6-hour incubation period, the transfection medium was exchanged for fresh complete medium. Cells were collected 48 to 72 hours after transfection for subsequent assays, such as RNA isolation for quantitative PCR (qPCR). To ensure experimental reproducibility, all procedures were conducted in triplicate.

### Cell proliferation assay

Cell proliferation of ccRCC cells under specified conditions was assessed using a CCK-8 assay kit (CK-04, Dojindo, Kumamoto, Japan) following the manufacturer’s protocol, as previously described. Briefly, prior to measurement, the culture medium was refreshed, and 10% (v/v) CCK-8 solution was added to each well. After incubation at 37 °C for 2 hours, absorbance was measured at 450 nm using a microplate reader (EXL800, BioTek Instruments, Winooski, VT, USA). Proliferation rates were normalized to the control group and expressed as percentages relative to control values.

### Invasion and migration assays

Cell invasion and migration were evaluated using transwell chambers (Millipore, Billerica, MA, USA) with or without Matrigel coating (BD Biosciences, NJ, USA), as previously reported. For the assay, 1×10^4^ 786-O or 769-P cells, or 1×10^5^ U937 cells, were suspended in serum-free RPMI-1640 medium and seeded into the upper chamber. The lower chamber contained RPMI-1640 medium supplemented with 20% fetal bovine serum (FBS) and conditioned medium (CM). After 36 or 48 hours of incubation, non-invading cells on the upper surface were gently removed with a cotton swab. Cells that migrated to the lower membrane surface were fixed with 4% paraformaldehyde (E672002, Sangon Biotech, Shanghai, China), stained with crystal violet (E607309, Sangon Biotech), and imaged at 200× magnification. Data are presented as mean ± standard deviation from three independent experiments.

### Statistical analysis

Numerical data are presented as the means ± standard deviations (SDs). Statistical differences between variables were assessed via a two-tailed Wilcoxon test. Survival curves were plotted via the Kaplan–Meier method and compared via log-rank analysis. Variables with p values < 0.1 in the univariate analysis were included in the multivariate Cox proportional hazards analysis. Differences were considered significant at p < 0.05. Time-dependent receiver operating characteristic (ROC) curve analyses were used to calculate the cutoff values of the H-scores of ERO1A and CD163 with SPSS 26.0 (IBM Corporation) software. The prognostic accuracy of combining ERO1A expression, CD163^+^ TAM density and other indicators was examined via Harrell’s concordance index (C-index). All the analyses were conducted using GraphPad Prism 9.5 software (GraphPad Software, Inc.) and SPSS 26.0 (IBM Corporation) software.

### Survival analysis

RNA-sequencing data and clinical information for kidney renal clear cell carcinoma (TCGA-KIRC) patients were obtained from TCGA database. The ERO1A expression data were log2-transformed. Cox proportional hazards regression was conducted to evaluate the associations of ERO1A expression with OS, DSS, and the PFI. Kaplan–Meier survival curves were plotted, and the log-rank test was used to assess the statistical significance of differences between groups.

## Results

### ERO1A expression is consistently upregulated in ccRCC

**Figure 1:**
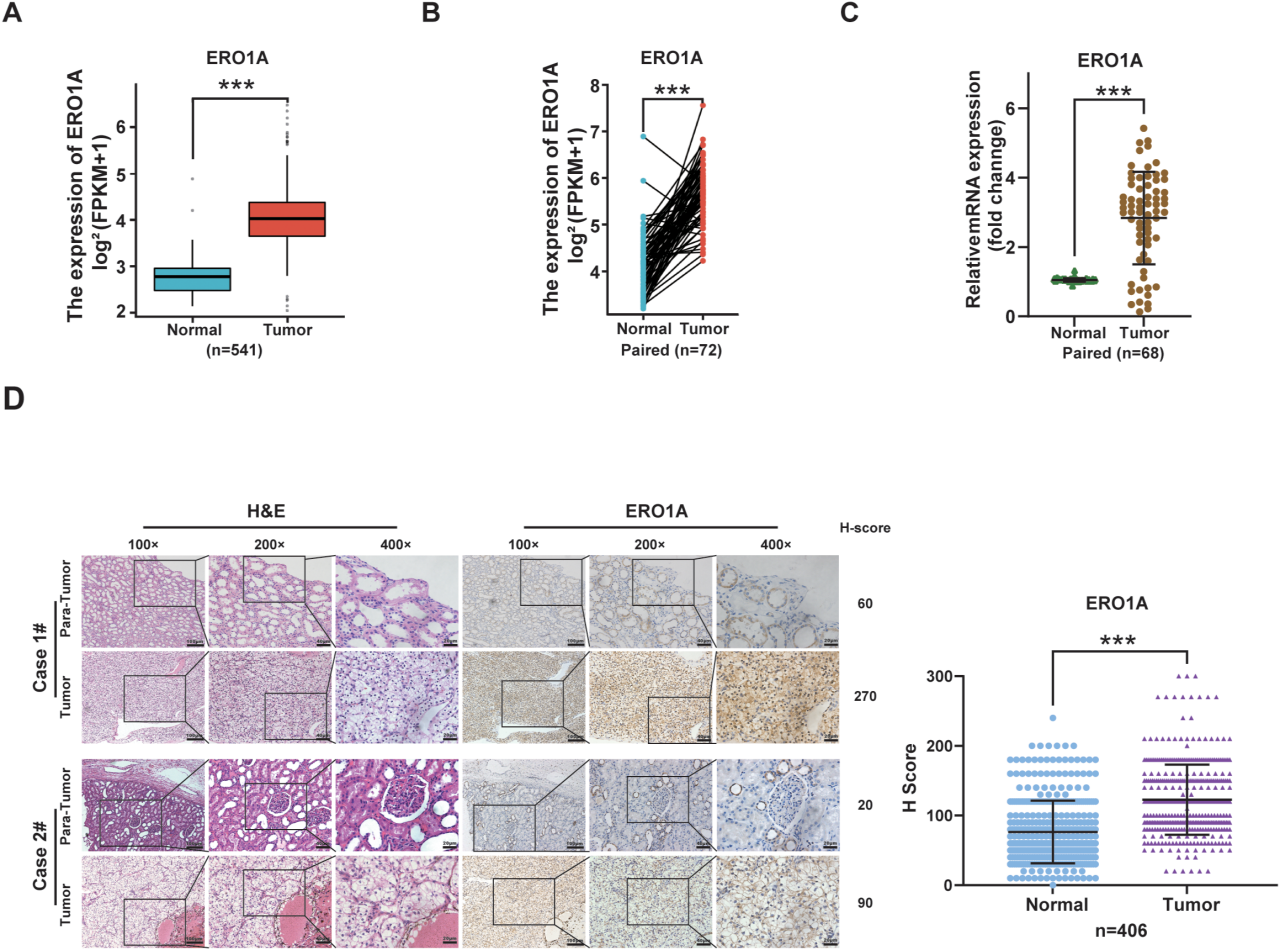
ERO1A expression is consistently upregulated in ccRCC. **(A)** The expression of *ERO1A* in ccRCC tissues and normal renal tissues (n = 541) was analyzed via TCGA database. **(B)** ERO1A expression was examined in ccRCC specimens and matched normal renal tissues (n = 72) from TCGA database. **(C)** The mRNA expression of *ERO1A* in paired ccRCC tissues and adjacent renal tissues was analyzed via real-time PCR (n = 68; ***p < 0.001). **(D)** Immunohistochemistry (IHC) was used to measure ERO1A expression in ccRCC tissues (n = 406; the different scale bars are shown in the images). Representative images of hematoxylin and eosin (H&E) and IHC staining are shown. H-scores were analyzed to compare the expression of ERO1A in paired ccRCC tissues with that in normal renal tissues, and the results are presented in the statistical chart (***p < 0.001). All p values are defined as ***p < 0.001, and the values are presented as the means ± SDs.

Analysis of ERO1A expression in ccRCC tissues using an online database (TCGA) revealed that *ERO1A* expression was higher in unpaired or paired ccRCC samples than in corresponding paracancerous samples (both *p* < 0.001; **Figures 1A, B**). Real-time PCR subsequently revealed that compared with the corresponding renal tissues, ccRCC tissues presented higher ERO1A expression (*p* < 0.001; **Figure 1C**). For validation, immunohistochemistry (IHC) was performed on ccRCC samples obtained from two hospitals (n = 406). Similar to the above findings, most ccRCC tissues presented high ERO1A expression (*p* < 0.001; **Figure 1D**), which was quantified according to the H score (refer to the *Materials and Methods* section for details). These findings demonstrated that ERO1A expression is consistently upregulated in ccRCC.

### High ERO1A expression predicts the malignant behavior of ccRCC

**Figure 2:**
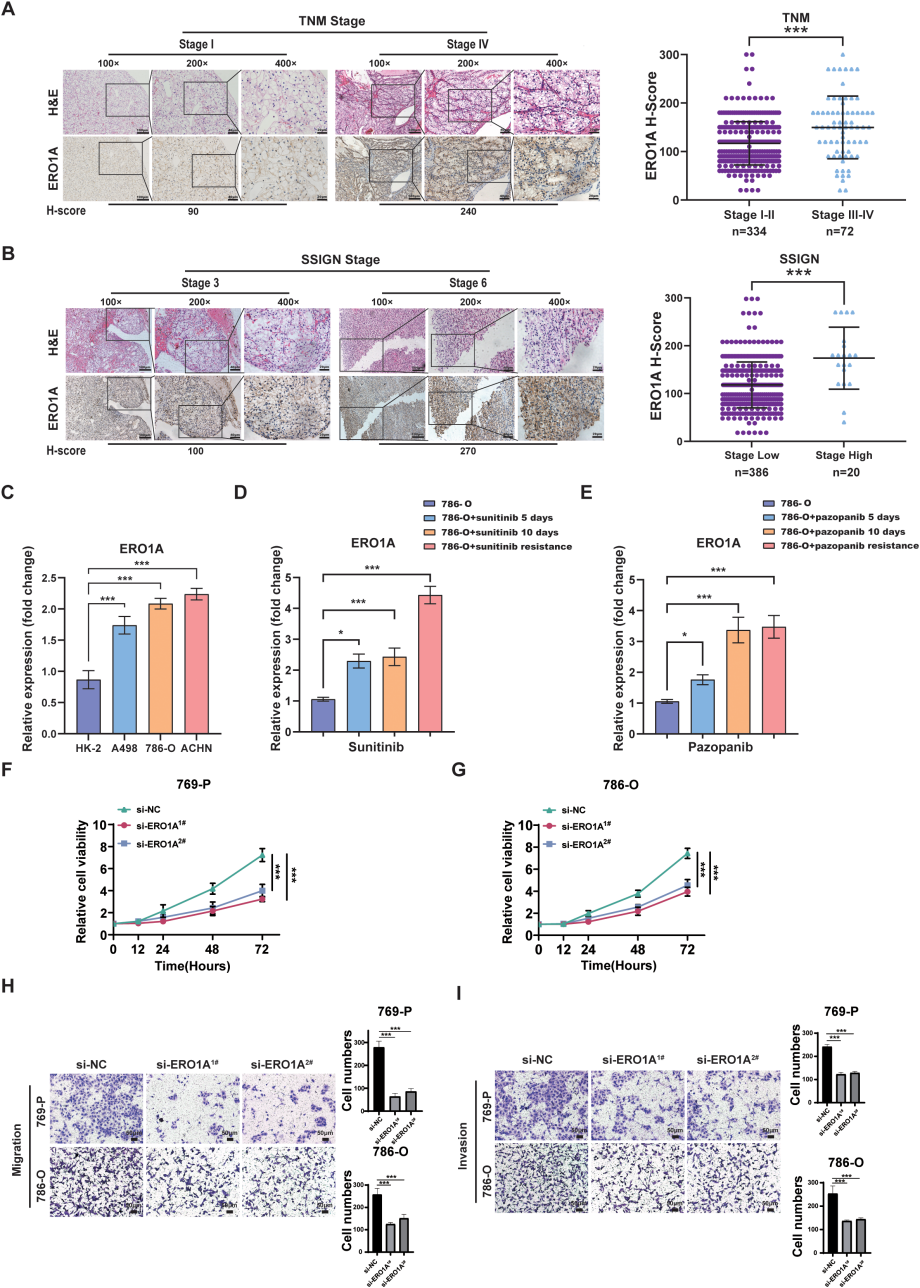
High ERO1A expression predicts ccRCC malignancy. **(A)** Representative pictures of H&E and IHC staining of ERO1A in ccRCC samples with different TNM stages (the different scale bars are shown in the images). **(B)** Representative images of H&E and IHC staining of ERO1A in ccRCC samples with different SSIGN scores (the different scale bars are shown in the images). **(C)** The mRNA expression of *ERO1A* was examined by real-time PCR in a normal renal cell line (HK-2) and ccRCC cell lines (A498, 786-O, and ACHN). **(D, E)**. Real-time PCR assays were used to detect *ERO1A* expression in sunitinib- and pazopanib-treated and resistant ccRCC cell lines (786-O–SR and 786-O–PR) compared with that in naïve 786-O cells. All p values are defined as *p < 0.05 and ***p < 0.001, and the values are presented as the means ± SDs. **(F, G)** CCK 8 assay was used to detect the proliferation of 769-P or 786-O cells without or with ERO1A knockdown. **(H, I)** Representative images and statistical analysis on the results of the migration invasion **(F)** or invasion **(I)** assays in 786-O or 769-P cells without or with ERO1A knockdown are shown (scale bar=50µm).

The relationship of ERO1A expression with malignant features of ccRCC was examined via IHC assays in ccRCC patients. As shown in **Figures 2A, B**, ERO1A expression was high in ccRCC samples with high TNM stages or SSIGN scores, whereas ERO1A expression was low in ccRCC samples with low TNM stages or SSIGN scores. Additionally, higher ERO1A expression was detected in ACHN cells (a metastatic ccRCC cell line) than in A498 or 786-O cells (nonmetastatic ccRCC cell lines) (**Figure 2C**). Elevated ERO1A expression was detected in ccRCC cell lines but not in HK-2 cells (a human renal tubular epithelial cell line) (**Figure 2C**). Moreover, compared with naïve cells, ccRCC cells treated with a targeted therapeutic drug (sunitinib or pazopanib) or resistant ccRCC cell lines presented higher ERO1A expression (**Figures 2D, E**). Therefore, these findings suggested that high ERO1A expression indicates malignant biological characteristics and strong resistance to targeted therapy in ccRCC. We next examined the biological function of ERO1A in ccRCC cells. First, siRNAs targeting ERO1A were employed in the 786-O and 769-P ccRCC cell lines (**Supplementary Figure S1A**). CCK-8 proliferation assay showed that compared with their respective control cells, ccRCC cells with ERO1A knockdown presented with decreased proliferation (**Figures 2F, G**). Moreover, ERO1A knockdown inhibited the invasion and migration abilities of ccRCC cells (**Figures 2H, I**).

### High ERO1A expression is associated with high tumor stage and poor prognosis for patients with ccRCC

**Figure 3:**
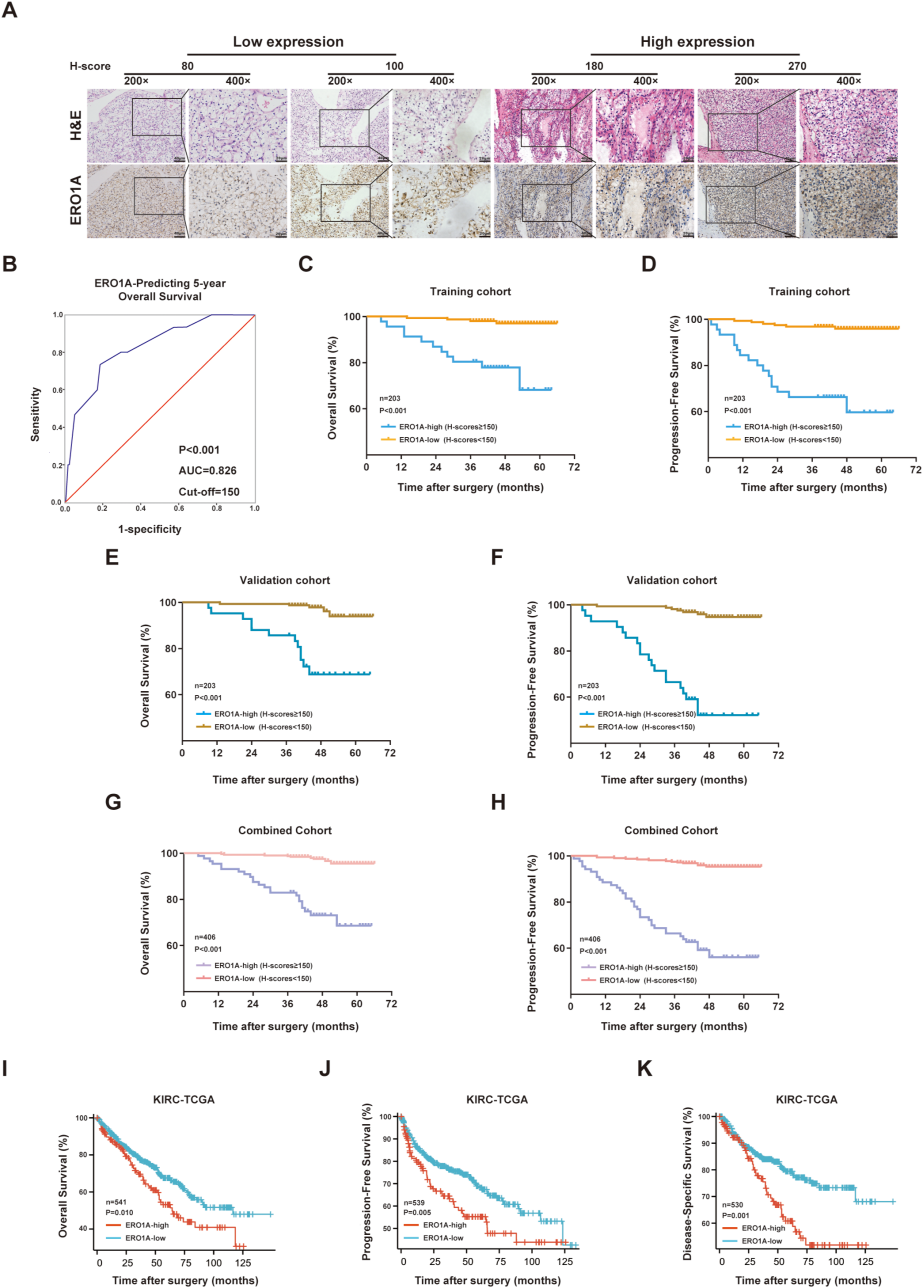
High ERO1A expression is associated with advanced tumor stage and poor prognosis in ccRCC patients. **(A)** Representative images of H&E and IHC staining of ERO1A in ccRCC tissues (the different scale bars are shown on the images). **(B)** Time-dependent ROC curve analysis was used to calculate the optimal H-score cutoff value of ERO1A in the training cohort (n = 203). **(C–H)**, Kaplan–Meier analyses of ccRCC patient OS and PFS were performed in the training cohort [n = 203; **(C, D)**], validation cohort [n = 203; **(E, F)**], and combined cohort [n = 406; **(G, H)**] (p value: log-rank test). **(I–K)**, Kaplan–Meier curves of OS, PFS, and DSS for ccRCC patients in TCGA-KIRC cohort grouped based on ERO1A expression level. All p values are defined as *p < 0.05, **p < 0.01 and ***p < 0.001, and the values are presented as the means ± SDs.

To investigate whether ERO1A serves as a useful prognostic marker, data from ccRCC patients from two hospitals were collected (cohort 1, n = 82; cohort 2, n = 324) (**Supplementary Table S1**). The ccRCC patients were randomly divided into training and validation cohorts. An IHC experiment was conducted to assess ERO1A expression in samples from ccRCC patients (**Figure 3A**). The optimal cutoff value was utilized in the training cohort to divide ccRCC patients into subgroups on the basis of high and low ERO1A expression (**Figure 3B**). With 5-year OS as the end point, time-dependent ROC curve analysis revealed that the optimal cutoff value was 150 (AUC = 0.826) (**Figure 3B**). In the training cohort, the TNM stages (*p* < 0.001) and SSIGN scores (*p* = 0.001) were higher in the ERO1A^high^ subgroup than in the ERO1A^low^ subgroup (**Table 1**). Moreover, Kaplan–Meier survival assays revealed that compared with the ERO1A^low^ subgroup, the ERO1A^high^ subgroup had shorter OS (*p* < 0.001) and PFS (*p* < 0.001) (**Figures 3C, D**). Furthermore, the cutoff value in the training cohort was applied to the validation or combined cohort. Compared with the ERO1A^low^ subgroup, the ERO1A^high^ subgroup presented a higher TNM stage and higher SSIGN score but shorter OS and PFS (**Figures 3E-H**; **Supplementary Tables S2, S3**). To further validate the above findings, data from TCGA were analyzed, which revealed a close correlation between ERO1A upregulation and poor ccRCC prognosis in terms of OS, PFS, and disease-specific survival (DSS) (**Figures 3I–K**). Therefore, these results suggested that ERO1A expressions is a significant indicator of ccRCC patient survival.

**Table 1:** Correlations between ERO1A expression and the clinicopathologic characteristics of patients with clear cell renal cell carcinoma in the training cohort (n = 203).

Characteristic	ERO1A		Sum (203)	P* value
	Low expression (n= 157)	High expression (n=46)		
Age				0.948
<60	93	27	120	
≥60	64	19	83	
Sex				0.157
Male	102	35	137	
Female	55	11	66	
TNM stage				<0.001
I−II	137	29	166	
III	20	17	37	
SSIGN				0.001*
0-4	156	41	197	
≥5	1	5	6	
CD163				0.012
LOW	110	23	133	
High	47	23	70	

*Statistical significance was calculated by the chi-square test or Fisher’s exact test for categorical/binary measures

### ERO1A expression in ccRCC is positively correlated with TAM density and predicts the survival period of ccRCC patients

**Figure 4:**
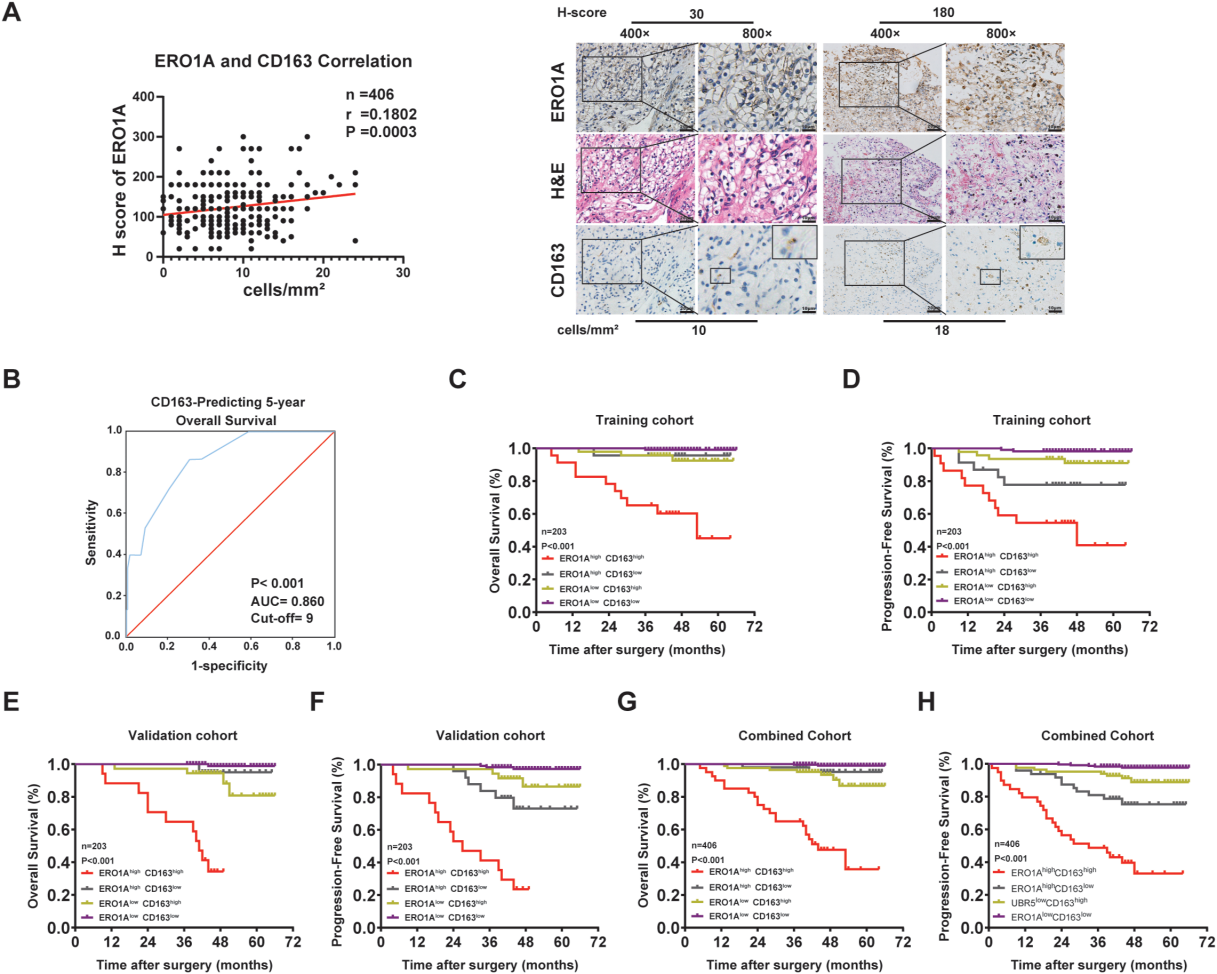
ERO1A expression in ccRCC tissues is positively correlated with TAM infiltration and is indicative of ccRCC prognosis. **(A)** Representative images of H&E and IHC staining of ERO1A and CD163 in ccRCC tissues (the different scale bars are shown in the images). Correlation analysis between the H-scores of ERO1A and CD163 in ccRCC tissues. **(B)** Time-dependent ROC curve analysis was employed to determine the optimal H-score cutoff value of CD163 in the training cohort (n = 203). **(C–H)**, Kaplan–Meier analyses of OS and PFS in ccRCC patients were performed in the training cohort [n = 203; **(C, D)**], validation cohort [n = 203; **(E, F)**], and combined cohort [n = 406; **(G, H)**] (p value: log-rank test). All p values are defined as *p < 0.05, **p < 0.01 and ***p < 0.001, and the values are presented as the means ± SDs.

The recurrence and metastasis of cancers are attributed to signal transduction pathways in malignant tumors and the regulatory network of interplay between cancer cells and tumor-infiltrating immune cells (TIICs) (22). To determine whether ERO1A expression is related to the infiltration of TIICs in ccRCC, IHC assays were performed to examine TIIC markers, such as CD163 (a TAM marker), CD8 (a marker for cytotoxic T lymphocytes, CTLs), and FOXP3 (a marker for regulatory T cells, Tregs), in ccRCC (23, 24) (**Figure 4A**; **Supplementary Figures S1B, C**). Correlation analysis revealed a positive correlation between ERO1A expression and CD163^+^ TAM density in ccRCC, but there was no correlation between ERO1A expression and the density of CD8^+^ CTLs or FOXP3^+^ Tregs (**Figure 4A**; **Supplementary Figures S1B, C**).

Because recent studies have demonstrated that combining intratumoral and TIIC markers increases the accuracy of survival prediction for ccRCC patients, we assessed whether the combination of ERO1A expression and CD163^+^ TAM density is beneficial for assessing ccRCC prognosis. Time-dependent ROC curves were constructed to determine the optimal cutoff values of ERO1A expression and CD163^+^ TAM density for classifying ccRCC patients in the training cohort (**Figures 3B**, **4B**). The ccRCC patients were classified into the following four subgroups: ERO1A^high^CD163^high^, ERO1A^low^CD163^low^, ERO1A^high^CD163^low^, and ERO1A^low^CD163^high^ (**Figures 4C-H**; **Supplementary Table S4**). As expected, the ERO1A^high^CD163^high^ subgroup had a higher TNM stage (p = 0.002) and SSIGN score (p = 0.002) but also shorter OS and PFS (both *p* < 0.001) (**Figures 4C, D**; **Supplementary Table S4**). Furthermore, the above results were confirmed in ccRCC patients from the validation and combined cohorts (**Figures 4E–H**; **Supplementary Table S5, S6**). These results indicated that combining ERO1A expression and CD163^+^ TAM density is helpful for predicting ccRCC patient disease development and survival rates.

### ERO1A and CD163^+^ TAM infiltration are independent risk factors for predicting the survival rate of patients with ccRCC

To conduct a more in-depth study of the ability of ERO1A expression and CD163^+^ TAM density to predict the survival rate of ccRCC patients, we assessed whether ERO1A expression and CD163^+^ TAM density are independent risk factors for assessing the survival rate of ccRCC patients via univariate and multivariate Cox regression analyses. As expected, ERO1A expression, CD163^+^ TAM density, TNM stage, and SSIGN score were confirmed to be independent risk factors for survival in ccRCC patients in the randomized training, validation, and combined cohorts, even after multivariable adjustment (**Table 2**; **Supplementary Tables S7, S8**). Therefore, these findings demonstrated that ERO1A expression and CD163^+^ TAM density serve as independent risk factors for predicting the survival rate of ccRCC patients.

**Table 2:** Univariate and multivariate Cox regression analyses of ERO1A expression, CD163 expression and clinicopathologic characteristics with overall survival and progression-free survival in the training cohort.

Characteristic		Overall survival				Progression free survival			
		Univariate		Multivariate		Univariate		Multivariate	
		HR (95% CI)	*P* Value	HR (95% CI)	*P* Value	HR (95% CI)	*P* Value	HR (95% CI)	*P* Value
Age (<60 vs. ≥60)	1.286 (0.466-3.547)		0.629			0.948 (0.628-1.432)	0.801		
Sex (Male vs. Female)	1.479 (0.525-4.169)		0.465			0.919 (0.378-2.237)	0.852		
TNM stage (I–II vs. III)	10.687 (3.644-31.341)		<0.001	6.924(1.820-26.340)	0.005	8.540 (3.688-19.776)	<0.001	4.330(1.612-11.629)	0.004
SSIGN (0-4 vs. ≥5)	48.536 (14.856-158.573)		<0.001	4.482(1.133-17.738)	0.033	37.545 (13.433-104.934)	<0.001	3.309 (1.026-10.667)	0.045
ERO1A expression (Low vs. High)	10.833 (3.446-34.060)		<0.001	3.656 (1.030-12.984)	0.045	12.316 (4.844-31.312)	<0.001	6.012 (2.163-16.706)	0.001
CD163 (Low vs. High)	13.218 (2.982-58.587)		0.001	11.316 (2.416-52.999)	0.002	4.779 (1.966-11.620)	<0.001	2.914 (1.117-7.604)	0.029

### Combining ERO1A expression and CD163^+^ TAM density is a superior indicator of the survival rate of ccRCC patients

Many studies, including our previous study, have indicated that integrating cancer markers, TIIC biomarkers, and TNM stage or the SSIGN score increases the accuracy of survival rate prediction for ccRCC patients (8). We next showed that the combination of ERO1A expression, CD163^+^ TAM density, and TNM stage or SSIGN score had a greater ability to predict the survival rate of ccRCC patients than any single marker alone. Time-dependent C-index assays were performed in the training cohort, which demonstrated that combining ERO1A expression and CD163^+^ TAM density increased the accuracy of the survival rate of ccRCC patients compared with any of the markers alone (**Table 3**). Furthermore, combining ERO1A expression, CD163^+^ TAM density, and TNM stage or SSIGN score resulted in the highest accuracy in evaluating ccRCC prognosis (**Table 3**), which was also validated in the validation and combined cohorts (**Table 3**). Taken together, these results indicated that combining ERO1A expression and CD163^+^ TAM density with TNM stage or SSIGN score increases the accuracy of predicting the prognosis of patients with ccRCC.

**Table 3:** C-index analysis of the prognostic accuracy of ERO1A, CD163 and other variables for overall survival and progression-free survival in the training, validation, and combined cohorts.

C-index (95% CI)	Overall survival Training cohort (n = 203)	Progression-free survival Training cohort (n = 203)	Overall survival Validation cohort (n = 203)	Progression-free survival Validation cohort (n = 203)	Overall survival Combined cohort (n = 406)	Progression-free survival Combined cohort (n = 406)
TNM stage	0.762 (0.618–0.905)	0.740 (0.617–0.864)	0.823 (0.702–0.944)	0.764 (0.651–0.877)	0.794 (0.700–0.887)	0.752 (0.669–0.836)
SSIGN	0.664 (0.493–0.835)	0.630 (0.491–0.770)	0.751 (0.599–0.903)	0.717 (0.590–0.843)	0.711 (0.596–0.825)	0.677 (0.583–0.771)
ERO1A	0.774 (0.640–0.908)	0.789 (0.680–0.898)	0.772 (0.642–0.903)	0.787 (0.681–0.892)	0.772 (0.679–0.866)	0.787 (0.711–0.863)
CD163	0.782 (0.672–0.891)	0.698 (0.582–0.813)	0.839 (0.743–0.935)	0.713 (0.600–0.825)	0.810 (0.738–0.883)	0.704 (0.623–0.784)
ERO1A+ CD163	0.868 (0.769–0.968)	0.850 (0.763–0.938)	0.907 (0.824–0.989)	0.847 (0.755–0.939)	0.887 (0.823–0.951)	0.847 (0.783–0.911)
ERO1A+ CD163+ TNM	0.903 (0.802–1.000)	0.869 (0.773–0.965)	0.958 (0.922–0.994)	0.904 (0.831–0.977)	0.932 (0.881–0.983)	0.887 (0.829–0.946)
ERO1A+ CD163+ SSIGN	0.887 (0.786–0.987)	0.873 (0.785–0.960)	0.937 (0.857–1.000)	0.893 (0.809–0.976)	0.914 (0.851–0.977)	0.883 (0.822–0.943)

## Discussion

Many studies have reported prognostic markers for ccRCC and have suggested that a single biomarker is not adequate for accurately predicting the prognosis of tumors. Owing to the heterogeneity of tumors, it is necessary to consider the tumor cells themselves and the tumor microenvironment in which tumors grow when studying tumors (25). An increasing number of studies have shown that combining tumor markers with TIIC markers in the cancer microenvironment more comprehensively predicts the prognosis of ccRCC patients (26, 27). Furthermore, the present study revealed that the combination of ERO1A expression, CD163^+^ TAM infiltration, and TNM stage or SSIGN score is more effective for assessing the survival rate of ccRCC patients.

ERO1A expression has been reported in several types of cancers, such as pancreatic ductal adenocarcinoma, colorectal cancer, lung cancer, and cholangiocarcinoma (16). Additionally, ERO1A is recognized as a prognostic factor in several types of cancers (16). Upregulated ERO1A expression in cancer patients is positively correlated with OS, recurrence-free survival (RFS), and disease-free survival (DFS). However, it remains unclear whether ERO1A expression is a prognostic indicator in ccRCC remains. The present study also revealed high ERO1A expression in most ccRCC samples. Moreover, high ERO1A expression in ccRCC patients indicated a poor prognosis, whereas low ERO1A expression was associated with a better prognosis. These findings indicated that ERO1A is an oncogene that is closely correlated with cancer prognosis.

Because cancer cells and the tumor microenvironment closely interact, we investigated whether ERO1A expression in cancers is related to the infiltration of TIICs in the tumor microenvironment. The tumor microenvironment consists of inflammatory cells, TIICs (including TAMs, T cells, and B cells), and their secreted factors (28). Recent studies have revealed that increased ERO1L expression supports the formation of an immunosuppressive tumor immune microenvironment through the recruitment of TAMs, Tregs and CTLs (28). Additionally, the inhibition of ERO1a and indoleamine 2,3-dioxygenase 1 (IDO1) facilitates monocyte infiltration and differentiation into dendritic cells to affect therapeutic responses in patients with pancreatic ductal adenocarcinoma (29). Furthermore, ERO1A is necessary for TAM-facilitated tumor cell invasion and increased MMP-9 expression (30). The present results also revealed that the expression level of ERO1A was positively correlated with CD163^+^ TAM infiltration in ccRCC, whereas ERO1A expression was not significantly correlated with the density of other TIICs in ccRCC. We also demonstrated that ERO1A expression and CD163^+^ TAM density were independent risk factors for predicting the survival rate of ccRCC patients. Therefore, these findings suggested that ERO1A expression in ccRCC affects the regulation of TIICs in the tumor microenvironment, thereby facilitating the formation of an immunosuppressive microenvironment suitable for ccRCC growth and progression.

At present, research on tumor prognostic markers has focused mainly on one or two markers, but this approach may not accurately predict tumor prognosis. Although TNM stage is used mostly as the basis for clinical treatment decision making in clinical practice, several key limitations of TNM staging exist. Increasing evidence indicates that tumor progression and therapeutic responsiveness are strongly influenced by complex interactions between cancer cells, immune cell subsets, and microenvironmental regulators, rather than by isolated molecular markers alone. In particular, recent studies have highlighted how tumor-intrinsic factors can reshape the immune microenvironment and modulate antitumor immunity, thereby affecting patient outcomes across multiple cancer types (31, 32). By considering both tumor factors and tumor microenvironment factors, the present study focused on clinical indicators commonly used to predict the survival rate of ccRCC patients. We investigated whether the combination of the ERO1A cancer marker and CD163^+^ TAM density in the tumor microenvironment with TNM stage or SSIGN score can be used to more accurately and comprehensively assess the survival rate of ccRCC patients. Specifically, the integrated ERO1A–CD163^+^ TAM–TNM stage (or SSIGN score) model more accurately predicted the survival rate of ccRCC patients than any single variable, such as ERO1A expression, CD163^+^ TAM density, TNM stage or SSIGN score.

The present study elucidated mainly the relationship between ERO1A expression and CD163^+^ TAM density in the tumor microenvironment of ccRCC, highlighting the importance of combining both with TNM stage or SSIGN score in predicting the survival rate of ccRCC patients. In the future, further confirmation with more ccRCC patients from multiple centers will be conducted to validate the postoperative significance of the integrated model. Furthermore, studies have reported that ERO1A reshapes the immune microenvironment of tumors, and the present study revealed that ERO1A expression is closely correlated with CD163^+^ TAM density. By situating these findings within the broader framework of tumor immunobiology, we underscore how ERO1A-driven immunoregulation may contribute to survival heterogeneity in ccRCC. Such insights not only bolster the biological plausibility of our model but also align with contemporary paradigms in cancer immunology, where tumor-immune interactions increasingly inform prognostic and therapeutic strategies. Therefore, we will explore in the future whether ERO1A facilitates the interaction between ccRCC cells and CD163^+^ TAMs and the related molecular mechanisms.

## Conclusions

Compared with the use of these markers alone, the combination of ERO1A expression and CD163^+^ TAM density with TNM stage or SSIGN score is more accurate for evaluating disease development and survival rate in ccRCC patients.

## Data Availability

The raw data supporting the conclusions of this article will be made available by the authors, without undue reservation.
