# Supplementary figures and images for: Combined assessment of ERO1A expression and CD163+ tumor-associated macrophage infiltration is superior to traditional assessment methods in predicting clear cell renal cell carcinoma prognosis
Source: Front Oncol. 2026 Feb 23;16:1732415. doi: 10.3389/fonc.2026.1732415 (PMC12968006; doi:10.3389/fonc.2026.1732415)

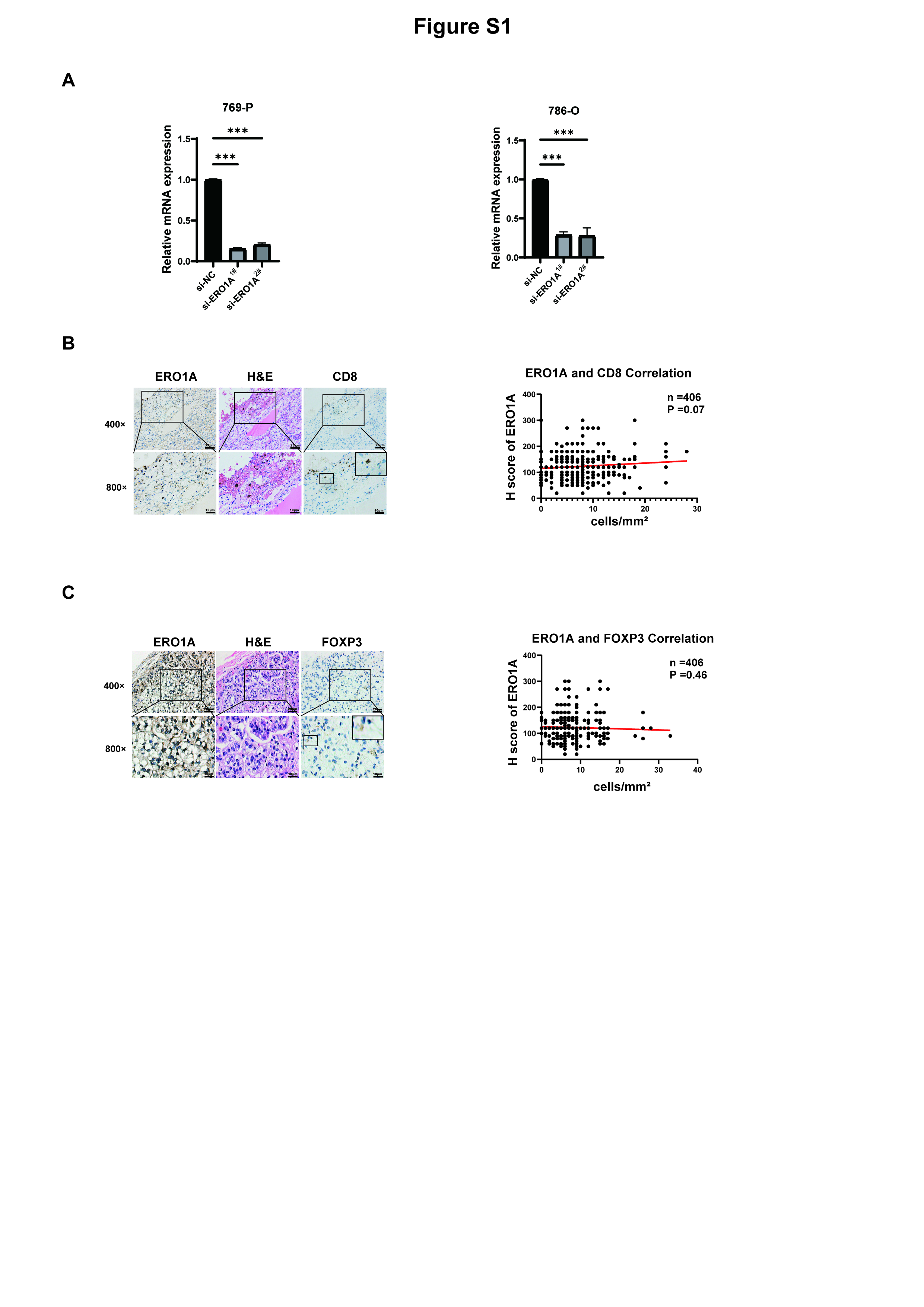

Supplement: Supplementary file 1 [file Image1.tif]
