# Supplementary material for: Combined assessment of ERO1A expression and CD163+ tumor-associated macrophage infiltration is superior to traditional assessment methods in predicting clear cell renal cell carcinoma prognosis
Source: Front Oncol. 2026 Feb 23;16:1732415. doi: 10.3389/fonc.2026.1732415 (PMC12968006; doi:10.3389/fonc.2026.1732415)
Supplement: Supplementary file 2 [file Table1.doc]

**Table S1 Demographics and characteristics of patients with clear cell renal cell carcinoma**

| Characteristic | training cohort  (n=203) | Validation cohort  (n=203) | Combined cohort  (n=406) |
| --- | --- | --- | --- |
| Age |  |  |  |
| <60 | 120 | 124 | 244 |
| ≥60 | 83 | 79 | 162 |
| Gender |  |  |  |
| Male | 137 | 142 | 279 |
| Female | 66 | 61 | 127 |
| TNM stage |  |  |  |
| I-II | 166 | 168 | 334 |
| III | 37 | 35 | 72 |
| SSIGN |  |  |  |
| 0-4 | 197 | 189 | 386 |
| ≥5 | 6 | 14 | 20 |
| ERO1A |  |  |  |
| Low expression | 157 | 161 | 318 |
| High expression | 46 | 42 | 88 |
| CD163 |  |  |  |
| Low expression | 133 | 150 | 283 |
| High expression | 70 | 53 | 123 |

**Table S2 The correlation between ERO1A expression and clinicopathologic characteristics of patients with clear cell renal cell carcinoma in the validation cohort (n = 203)**

|  | ERO1A | |  |  |
| --- | --- | --- | --- | --- |
| Characteristic | Low expression  (n=161) | High expression  (n=42) | Sum  (203) | P* value |
| Age |  |  |  | 0.556 |
| <60 | 100 | 24 | 124 |  |
| ≥60 | 61 | 18 | 79 |  |
| Gender |  |  |  | 0.369 |
| Male | 115 | 27 | 142 |  |
| Female | 46 | 15 | 61 |  |
| TNM stage |  |  |  | <0.001 |
| Ⅰ-Ⅱ | 141 | 27 | 168 |  |
| Ⅲ | 20 | 15 | 35 |  |
| SSIGN |  |  |  | <0.001* |
| 0-4 | 156 | 33 | 189 |  |
| ≥5 | 5 | 9 | 14 |  |
| CD163 |  |  |  | 0.017 |
| LOW | 125 | 25 | 150 |  |
| High | 36 | 17 | 53 |  |

*Statistical significance was calculated by chi-square test or fisher’s exact test for categorical/binary measures

**Table S3. The correlation between ERO1A expression and clinicopathologic characteristics of patients with clear cell renal cell carcinoma in the combined cohort (n = 406)**

|  | ERO1A | |  |  |
| --- | --- | --- | --- | --- |
| Characteristic | Low expression  (n=318) | High expression  (n=88) | Sum  (406) | P* value |
| Age |  |  |  | 0.643 |
| <60 | 193 | 51 | 244 |  |
| ≥60 | 125 | 37 | 162 |  |
| Gender |  |  |  | 0.692 |
| Male | 217 | 62 | 279 |  |
| Female | 101 | 26 | 127 |  |
| TNM stage |  |  |  | <0.001 |
| Ⅰ-Ⅱ | 278 | 56 | 334 |  |
| Ⅲ | 40 | 32 | 72 |  |
| SSIGN |  |  |  | <0.001* |
| 0-4 | 312 | 74 | 386 |  |
| ≥5 | 6 | 14 | 20 |  |
| CD163 |  |  |  | <0.001 |
| LOW | 235 | 48 | 283 |  |
| High | 83 | 40 | 123 |  |

*Statistical significance was calculated by chi-square test or fisher’s exact test for categorical/binary measures

**Table S4. The correlation between expressions of ERO1A, CD163 and clinicopathologic characteristics of patients with clear cell renal cell carcinoma in the training cohort (n=203)**

|  | ERO1A/ CD163 expression | |  |  |  |
| --- | --- | --- | --- | --- | --- |
| Characteristic | ERO1A^low^  CD163^low^  (n=110) | ERO1A^high^  CD163^low^  (n=23) | ERO1A^low^  CD163^high^  (n=47) | ERO1A^high^  CD163^high^  (n=23) | P* value |
| Age |  |  |  |  | 0.521 |
| <60 | 65 | 16 | 28 | 11 |  |
| ≥60 | 45 | 7 | 19 | 12 |  |
| Gender |  |  |  |  | 0.175 |
| Male | 76 | 18 | 26 | 17 |  |
| Female | 34 | 5 | 21 | 6 |  |
| TNM stage |  |  |  |  | 0.002 |
| Ⅰ-Ⅱ | 94 | 15 | 43 | 14 |  |
| Ⅲ | 16 | 8 | 4 | 9 |  |
| SSIGN |  |  |  |  | 0.002* |
| 0-4 | 110 | 22 | 46 | 19 |  |
| ≥5 | 0 | 1 | 1 | 4 |  |

*Statistical significance was calculated by chi-square test or fisher’s exact test for categorical/binary measures

**Table S5 The correlation between expressions of ERO1A, CD163 and clinicopathologic characteristics of patients with clear cell renal cell carcinoma in the validation cohort(n=203)**

|  | ERO1A/ CD163 expression | |  |  |  |
| --- | --- | --- | --- | --- | --- |
| Characteristic | ERO1A^low^  CD163^low^  (n= 125) | ERO1A^high^  CD163^low^  (n= 36) | ERO1A^low^  CD163^high^  (n= 25) | ERO1A^high^  CD163^high^  (n= 17) | P* value |
| Age |  |  |  |  | 0.455 |
| <60 | 75 | 25 | 16 | 8 |  |
| ≥60 | 50 | 11 | 9 | 9 |  |
| Gender |  |  |  |  | 0.384 |
| Male | 86 | 29 | 17 | 10 |  |
| Female | 39 | 7 | 8 | 7 |  |
| TNM stage |  |  |  |  | ＜0.001 |
| Ⅰ-Ⅱ | 113 | 28 | 19 | 8 |  |
| Ⅲ | 12 | 8 | 6 | 9 |  |
| SSIGN |  |  |  |  | <0.001* |
| 0-4 | 122 | 34 | 23 | 10 |  |
| ≥5 | 3 | 2 | 2 | 7 |  |

*Statistical significance was calculated by chi-square test or fisher’s exact test for categorical/binary measures

**Table S6. The correlation between expressions of ERO1A, CD163 and clinicopathologic characteristics of patients with clear cell renal cell carcinoma in the combined cohort (n=406)**

|  | ERO1A/ CD163 expression | |  |  |  |
| --- | --- | --- | --- | --- | --- |
| Characteristic | ERO1A^low^  CD163^low^  (n= 235) | ERO1A^high^  CD163^low^  (n= 83) | ERO1A^low^  CD163^high^  (n= 48) | ERO1A^high^  CD163^high^  (n= 40) | P* value |
| Age |  |  |  |  | 0.259 |
| <60 | 140 | 53 | 32 | 19 |  |
| ≥60 | 95 | 30 | 16 | 21 |  |
| Gender |  |  |  |  | 0.883 |
| Male | 162 | 55 | 35 | 27 |  |
| Female | 73 | 28 | 13 | 13 |  |
| TNM stage |  |  |  |  | <0.001 |
| Ⅰ-Ⅱ | 207 | 71 | 34 | 22 |  |
| Ⅲ | 28 | 12 | 14 | 18 |  |
| SSIGN |  |  |  |  | <0.001* |
| 0-4 | 232 | 80 | 45 | 29 |  |
| ≥5 | 3 | 3 | 3 | 11 |  |

*Statistical significance was calculated by chi-square test or fisher’s exact test for categorical/binary measures

**Table S7 Univariate and multivariate cox regression analysis of ERO1A, CD163 and clinicopathologic characteristics**

**with overall survival and progression-free survival in the validation cohort**

|  | | Overall survival | | | | |  | Progression free survival | | | | |
| --- | --- | --- | --- | --- | --- | --- | --- | --- | --- | --- | --- | --- |
|  | | Univariate | |  | Multivariate | |  | Univariate | |  | Multivariate | |
| Characteristic | | HR (95% CI) | *P* Value |  | HR (95% CI) | *P* Value |  | HR (95% CI) | *P* Value |  | HR (95% CI) | *P* Value |
| Age (<60 vs ≥60) | 0.564(0.217-1.461) | | 0.239 |  |  |  |  | 0.757 (0.519-1.105) | 0.150 |  |  |  |
| Gender (Male vs Female) | 2.111(0.813-5.477) | | 0.132 |  |  |  |  | 1.594 (0.740-3.435) | 0.243 |  |  |  |
| TNM stage ( Ⅰ-Ⅱ vs Ⅲ) | 18.009(5.864-55.307) | | <0.001 |  | 5.688 (1.735-18.650) | 0.004 |  | 10.778 (4.926-23.585) | <0.001 |  | 3.901 (1.586-9.596) | 0.003 |
| SSIGN (0-4 vs ≥5) | 22.632(8.587-59.649) | | <0.001 |  | 3.734 (1.101-12.662) | 0.034 |  | 20.392 (9.346-44.495) | <0.001 |  | 2.883 (1.074-7.740) | 0.036 |
| ERO1A expression (Low vs High) | 10.704(3.766-30.422) | | <0.001 |  | 3.838 (1.094-13.468) | 0.036 |  | 11.984 (5.232-27.450) | <0.001 |  | 5.897 (2.365-14.702) | <0.001 |
| CD163 ( LOW vs High) | 23.650(5.407-103.441) | | <0.001 |  | 14.135 (3.135-64.414) | 0.001 |  | 5.490 (2.513-11.994) | <0.001 |  | 2.407 (1.019-5.689) | 0.045 |

**Table S8 Univariate and multivariate cox regression analysis of ERO1A, CD163 and clinicopathologic characteristics with**

**overall survival and progression-free survival in the combined cohort**

|  | | Overall survival | | | | |  | Progression free survival | | | | |
| --- | --- | --- | --- | --- | --- | --- | --- | --- | --- | --- | --- | --- |
|  | | Univariate | |  | Multivariate | |  | Univariate | |  | Multivariate | |
| Characteristic | | HR (95% CI) | *P* Value |  | HR (95% CI) | *P* Value |  | HR (95% CI) | *P* Value |  | HR (95% CI) | *P* Value |
| Age (<60 vs ≥60) | 0.808 (0.571-1.143) | | 0.230 |  |  |  |  | 0.842 (0.638-1.112) | 0.228 |  |  |  |
| Gender (Male vs Female) | 1.810 (0.899-3.645) | | 0.103 |  |  |  |  | 1.247 (0.700-2.223) | 0.459 |  |  |  |
| TNM stage ( Ⅰ-Ⅱ vs Ⅲ) | 13.946 (6.449-30.157) | | <0.001 |  | 6.341 (2.692-14.938) | <0.001 |  | 9.678 (5.459-17.155) | <0.001 |  | 4.321 (2.244-8.321) | <0.001 |
| SSIGN (0-4 vs ≥5) | 26.328 (12.791-54.194) | | <0.001 |  | 3.404 (1.442-8.034) | 0.005 |  | 21.511 (11.856-39.030) | <0.001 |  | 2.744 (1.338-5.627) | 0.006 |
| ERO1A expression (Low vs High) | 10.900 (5.039-23.582) | | <0.001 |  | 4.062 (1.710-9.650) | 0.001 |  | 12.052 (6.488-22.385) | <0.001 |  | 6.098 (3.110-11.957) | <0.001 |
| CD163 ( LOW vs High) | 17.594 (6.171-50.164) | | <0.001 |  | 12.175 (4.188-35.398) | <0.001 |  | 5.001 (2.785-8.981) | <0.001 |  | 2.734 (1.465-5.104) | 0.002 |
